# School nurses’ attitudes towards and experiences of the Swedish school-based HPV vaccination programme – A repeated cross sectional study

**DOI:** 10.1371/journal.pone.0175883

**Published:** 2017-04-18

**Authors:** Maria Grandahl, Margareta Larsson, Tanja Tydén, Christina Stenhammar

**Affiliations:** 1 Department of Women’s and Children’s Health, Uppsala University, Uppsala, Sweden; 2 Department of Public Health and Caring Sciences, Uppsala University, Uppsala, Sweden; Rudjer Boskovic Institute, CROATIA

## Abstract

The aim was to investigate school nurses’ attitudes towards, and experiences of vaccination against human papillomavirus (HPV), and compare the results with a similar study three years earlier. School nurses (n = 736) from all counties in Sweden completed a questionnaire in spring 2016, four years after the implementation of the national HPV vaccination programme, and three years after the previous survey. Overall, the school nurses had more favourable attitudes towards the HPV vaccination programme compared to the study in 2013 (*p* = 0.015). More than half of the nurses (n = 415, 56%) strongly agreed that boys should also be offered the vaccine (*p*<0.001). There were no differences in school nurses’ perceived knowledge about HPV in order to inform and to answer questions about the vaccine from the girls or from the parents. More than half of the nurses (n = 409, 56%) reported that they needed more education about HPV. Almost all nurses (n = 659, 90%) had been contacted by parents with questions about the vaccine, and most questions were related to vaccine safety. School nurses have a more favourable attitude towards the vaccination programme against HPV compared to three years earlier, although almost all nurses had been contacted by parents with diverse questions and concerns. The nurses believed that they needed more education about HPV. Thus, it is essential to provide ongoing education and training for school nurses who are key healthcare professionals for providing information about HPV and HPV vaccination to parents and to pupils.

## Introduction

An infection caused by high-risk human papillomavirus (HPV) is a major cause to the global burden of HPV-related cancer [1]. The most common high-risk HPV types can effectively be prevented by vaccination against HPV, and vaccination programmes are implemented worldwide. The coverage is suboptimal in many countries [2], and vaccine hesitancy is a growing challenge globally [3, 4]. The uptakes vary from 1% in Japan to about 27% in Denmark to 50% (boys)– 60% (girls) in the US. School-based vaccination programmes generally have higher uptake, ranging between 70% in Australia and more than 85% in the UK and over 90% of the school girls in Rwanda [2]. High coverage is essential to receive herd immunity, and it is imperative to increase coverage rates in settings where coverage remains low.

The quadrivalent HPV vaccine was included in the national vaccination programme for children in Sweden in 2012. The vaccine is primarily offered free of charge to girls aged 10–12. The vaccine is delivered in school health services and provided by school nurses. The national coverage is about 80% for the first dose [5], and the national aim of over 90% coverage has not yet been reached. To promote higher vaccine uptake, catch-up vaccination is also offered in school health for girls who have missed previous opportunities up to age 18.

There is an agreement that healthcare providers have an important role in the success of the vaccination programmes [6–9]. Studies on school nurse attitudes and beliefs [10–13] indicate that nurses are in favour of HPV vaccination although it is time-consuming and the logistics are a challenge. Hudson et al. [14] and Perkins [15] emphasise the importance of healthcare providers’ communication skills in addressing parents’ vaccine hesitancy. Qualitative studies [13, 16, 17] undertaken among school nurses in a similar setting, in the United Kingdom, show that school nurses have a unique position in informing parents and addressing their questions and concerns. Rosen et al. [10] have highlighted the importance of school nurses’ need for adequate resources and knowledge of HPV.

In a population based study [18] among all Swedish school nurses vaccinating against HPV during the early start of the national vaccination programme in 2013, most nurses were in favour of the vaccination programme. However, the majority had experienced difficulties. Most nurses had been contacted directly by parents who had concerns about the vaccine, especially regarding vaccine safety and effectiveness. One in five girls is not vaccinated against HPV in Sweden, and healthcare providers have an important role if the vaccination programme is to be successful. The aim was thus to investigate school nurses’ attitudes towards and experiences of HPV vaccination—a repeated study after its implementation in Sweden. It was hypothesised that school nurses had more favourable attitudes towards the vaccination programme, perceived less barriers with the vaccinations and had higher level of perceived knowledge about HPV vaccine compared to our previous study [18] at the start of the national vaccination programme against HPV.

## Methods

### Ethical considerations

The study was performed according to the Declaration of Helsinki, and the national guidelines [19] and the Regional Ethical Review Board in Uppsala, Sweden, D.nr. 2012/455 approved the study.

### Study design and sample

We carried out a repeated population-based cross sectional study among school nurses in Sweden between March and June 2016. Those eligible for inclusion were school nurses who participated in the national school-based HPV vaccination programme. The study follows reports of cross-sectional studies according to STROBE (S1 File) [20].

Sweden has a population of about 10 million inhabitants, 17% are born in another country, and over 22% have an immigrant background (both parents born outside Sweden). Sweden is divided into 21 counties and 290 municipalities. The counties are accountable for healthcare, hospital and primary care, and the municipalities are responsible for school health services including providing vaccinations to children aged 6–18. The school health services include the school nurse and the school doctor. The school nurse is present on a weekly basis, while the school doctor attends the school occasionally. School nurses are delegated the responsibility for all aspects of vaccinations including providing information to parents and children, parental consent, logistics, documentation and administration of the vaccine. Most nurses work alone, but for patient safety, they are recommended to work in pairs when vaccinating. School nurses in neighbouring schools often provide assistance; they “help each other out”.

### Procedure

We used a similar procedure as in the 2013 study [18] and recruited school nurses via the official website of the Swedish Association for School Nurses (www.skolskoterskor.se). The authors (MG and CS) sent emails to head nurses in each municipality (n = 290) regarding the study’s aim and procedure and requested that they inform their school nurses who vaccinate against HPV about the invitation to the study. On the website, school nurses were informed that the study was voluntary and that the responses were confidential. Those who agreed to participate were asked to complete the questionnaire. No written informed consent was obtained since the data were analysed anonymously. A reminder was sent to the head nurses after one month. In addition, we called the head nurses to confirm that they had received our email with information about the study. We were aware of school nurses’ high workload at this time and also provided information about the study at the National Conference for School nurses in April 2016. About half of all school nurses in Sweden attend this national conference. Nurses vaccinating against HPV, and who were interested in participating could complete the questionnaire during the conference.

### Questionnaire

The questionnaire was based on our previous study [18] and included 26 questions. The main part comprised questions about school nurses’ attitudes towards and perceived knowledge of HPV and HPV vaccination. The questions had multiple-choice alternatives, with four-point scales, ranging from “strongly agree” to “strongly disagree”. The questionnaire also comprised questions about information provided to parents and pupils by the school nurse. Also included were three open-ended questions regarding parents’ most frequently asked questions, school nurses’ experiences of why parents decline the vaccination, and if the nurse´s had perceived barriers with the HPV vaccination. Finally, the questionnaire comprised background demographic data. It took about 15 minutes to complete the questionnaire.

### Data analysis

Categorical and ordinal data are presented as frequencies and percentages, n (%), and continuous data as means and standard deviations. Differences between the groups of nurses in 2013 and in 2016, respectively, were calculated using Mann-Whitney *U* test for variables on ordinal level, and Spearman’s χ^2^ was used for variables on nominal level. Data (S2 File. Data set 2016 and S3 File. Data set 2013) were analysed using SPSS (22.0) software (SPSS Inc., 2013), and p values of <.05 were considered statistically significant. The open-ended questions were analysed by content analyses [21], i.e. systematically organising and categorising the answers.

The index, “overall HPV attitude”, included all items regarding school nurses’ attitudes towards HPV. The index was created by grouping together “strongly agree” and “agree partially” to “agree”, while “strongly disagree” and “disagree partially” were grouped together to the new variable “disagree”. Thereafter, all items for “HPV attitude” were added together to form the overall HPV attitude, ranging from 0 to 1 for each item (total scores = 0–10 points). Differences in attitudes between 2013 and 2016 were analysed with Mann- Whitney *U* test.

The definition of a favourable attitude towards HPV vaccination was that the school nurse agreed, strongly or partly, that it was appropriate for HPV vaccination to be introduced in the general childhood vaccination programme and that school nurses were responsible for these vaccinations.

## Results

In total, 736 school nurses from all 21 counties completed the questionnaire (see Table 1). Of those, 367 nurses completed the questionnaire at the National Conference for school nurses in April 2016. The participants represented the majority of all Swedish municipalities, 204/290 (70.3%) and included diverse schools situated in different socioeconomic regions, both rural and urban. The nurses worked at both public and private schools with a varying number of pupils. Almost nine out of ten nurses (87%) had at least one year of specialist training education, mainly in public health or paediatrics (Table 1).

**Table 1:** Characteristics of the participating school nurses in 2016 (n = 736) and in 2013 (n = 851).

Characteristics	School nurses 2016 mean (SD)	School nurses 2013 mean (SD)
**Age, years**	51.4 (8.5)	51.7 (7.5)
**Years as a school nurse**	10.0 (7.8)	10.17 (7.3)
	**n (%)**	**n (%)**
**Sex**		
Women	715 (97.1)	840 (98.7)
Men	7 (1.0)	11 (1.3)
Other/not stated	8 (1.1)	-
**Specialist Nursing Education**		
Public health/District nursing	336 (45.7)	411 (48.3)
Paediatric and adolescent health	225 (30.6)	317 (37.3)
School health	53 (7.2)	74 (8.7)
Other (intensive care, anaesthesia or midwifery)	25 (3.4)	40 (4.7)
None	21 (2.9)	9 (1.1)
**Workplace**		
Public school	650 (88.3)	781 (91.8)
Private school	70 (9.5)	70 (8.2)
**The school´s geographical area**		
Urban	337 (45.8)	296 (34.8)
Rural	194 (26.4)	209 (24.6)
City	191 (26.0)	346 (40.6)

### Main results compared to the study in 2013

The participating school nurses were similar to the participants in 2013 regarding background characteristics, although more school nurses reported working in schools situated in a city. However, it should be noted, that in Sweden, there is sometimes a fine line between urban area and a city.

The participants had more favourable attitudes overall towards the national HPV vaccination programme, and fewer nurses had experienced difficulties compared with our study in 2013. Almost all nurses had been contacted by parents who had questions about the vaccine, and most questions were related to vaccine safety. There were no significant differences in school nurses’ perceived knowledge about HPV in order to inform and to answer questions about the vaccine, although over half of the nurses believed that they needed more education about HPV.

### Attitudes towards HPV vaccination

School nurses attitudes towards HPV vaccination in 2016 compared to 2013 are presented in Table 2. Overall, the results indicate more favourable attitudes (p = 0.015). There were significant differences in all items, except *HPV vaccination may improve awareness of sexually transmitted infections* (see Table 2). More school nurses strongly agreed (86% in 2016 vs 64% in 2013) that it was appropriate for HPV vaccination to be introduced into the general childhood vaccination programme. Over half of the school nurses strongly agreed (56% in 2016 vs 41% in 2013) that boys should also be offered the HPV vaccine. Fewer (16% in 2016 vs 27% in 2013) considered that HPV vaccine was more painful than other vaccinations, and fewer school nurses strongly agreed (18% in 2016 vs 23% in 2013) that fear of needles is a problem. However, more than four out of ten nurses (45%) agreed partially to this statement. More nurses strongly agreed (38% in 2016 vs 32% in 2013) that those who declined vaccination should be offered the vaccination at a later date (Table 2).

**Table 2:** School nurses’ attitudes towards HPV vaccination.

	Strongly agree n (%)		Partially agree n (%)		Partially disagree n (%)		Strongly disagree n (%)		p-value†
	2016 (n = 736)	2013 (n = 851)	2016 n = (736)	2013 (n = 851)	2016 n = (736)	2013 (n = 851)	2016 n = (736)	2013 (n = 851)	
It is appropriate that HPV vaccination was introduced in the general childhood vaccination programme	629 (85.5)	544 (63.9)	88 (12.0)	243 (28.6)	13 (1.8)	53 (6.2)	2 (0.3)	11 (1.3)	<.001
Boys should also be offered the HPV vaccine in the school based vaccination programme	415 (56.4)	352 (41.4)	234 (31.8)	341 (40.1)	50 (6.8)	113 (13.3)	26 (3.5)	45 (5.3)	<.001
HPV vaccine is more painful than other vaccinations	115 (15.6)	232 (27.3)	247 (33.6)	302 (35.5)	120 (16.3)	172 (20.2)	244 (33.2)	145 (17.0)	<.001
Fear of needles is a problem	132 (17.9)	198 (23.3)	328 (44.6)	375 (44.1)	158 (21.5)	176 (20.7)	108 (14.7)	102 (12.0)	0.012
Those who declined vaccination should be offered the vaccination later	278 (37.8)	271 (31.8)	234 (31.8)	238 (28.0)	120 (16.3)	184 (21.6)	98 (13.3)	158 (18.6)	<.001
I have sufficient confidence in the authorities’ decisions about HPV vaccination	491 (66.7)	393 (46.2)	197 (26.8)	356 (41.8)	27 (3.7)	81 (9.5)	12 (1.2)	21 (2.5)	<.001
HPV vaccination may result in decreased condom use	34 (4.6)	54 (6.3)	271 (36.8)	377 (44.3)	216 (29.3)	257 (30.2)	203 (27.6)	163 (19.2)	<.001
HPV vaccination may result in increased number of sexual partners	12 (1.6)	31 (3.6)	138 (18.8)	167 (19.6)	235 (31.9)	335 (39.4)	334 (45.4)	318 (37.4)	0.002
HPV vaccination may improve the awareness of sexually transmitted infections	193 (26.2)	199 (23.4)	407 (55.3)	527 (61.9)	103 (14.0)	95 (11.2)	20 (2.7)	30 (3.5)	0.593
HPV vaccination may reduce participation in Pap smear tests	37 (5.0)	72 (8.5)	310 (42.1)	466 (54.8)	236 (32.1)	227 (26.7)	138 (18.8)	86 (10.1)	<.001

^†^Mann-Whitney *U* test

A smaller proportion of school nurses believed that HPV vaccination might result in decreased condom use, 41% agreed strongly or partially vs 51% in 2013. There were also fewer nurses (21% in 2016 vs 23% in 2013) who believed that HPV vaccination might result in increased number of sexual partners. In addition, more nurses (67% in 2016 vs 46% in 2013) agreed strongly that they had confidence in decisions made by authorities regarding HPV vaccination (Table 2).

### Experiences- perceived barriers

There were significant differences regarding what parents express to the nurses for declining the vaccine (p < .001). A higher proportion of the nurses reported that parents were concerned of side effects (n = 430, 58.4% in 2016 vs n = 459, 53.9% in 2013). A total of 236 nurses reported on the girl’s young age (32.1% vs n = 297, 34.9% in 2013), while 25 nurses (3.4%) reported religious reasons compared with 34 nurses (4.0%) in 2013. Moreover, thirteen nurses (2%) believed fear of needles was the main reason, two nurses (0.3%) reported the girl’s medical condition (other disease), and six nurses (1%) reported do not know.

In the open-ended questions, fewer nurses (n = 366, 50%) reported difficulties with the vaccination compared to in 2013 (n = 570, 67%). In the content analysis of the open-ended questions, four categories emerged: 1) *fear of needles and pain* included fainting and dizziness (n = 135, 37%); 2) *logistical problems* included workload, missing parental consent and language difficulties (n = 65, 18%); 3) *fear of vaccine side-effects and questioning from parents* included negative media reports (n = 53, 15%); and 4) *girls young age* (n = 13, 4%). Some representative statements are presented below.

‘At this age, many are, more or less, afraid of vaccination. Pupils influence each other’.

‘The second vaccination is sometimes difficult because many think the first was very painful—some have become afraid of needles in between the vaccinations’.

‘With all the vaccinations, and also HPV, it is difficult to get all the consent forms. It’s a lot of administrative work to send, collect, return, call parents, plan the day, reschedule those who were ill, etc. … Parents are invited to be present. Sometimes parents are present’.

‘Before the alarm [the reports of side effects in Denmark] last year, I vaccinated 99%, after that, it is only 50%.

‘[It is a challenge] to get the right information to the guardians at the right time before or between media scaremongering. Those who refuse socialise with each other and it becomes more difficult to influence’.

‘Some parents have, through social media, conveyed their position that HPV vaccination is not good based on their view that the vaccine can cause severe side effects. I also feel that following the vaccination against swine flu cases with cases of severe side effects, it has influenced some parents' position not to vaccinate’.

### Perceived knowledge about HPV vaccine

School nurses perceived knowledge about HPV vaccine is presented in Table 3. There were no significant differences in school nurses’ perceived knowledge about HPV in order to inform and to answer questions about the vaccine in 2016 compared with the results in 2013 (Table 3). In the additional questions in the 2016 survey regarding knowledge of HPV vaccine, more than one-third (38%) agreed strongly that they had good knowledge about the safety profile of HPV vaccine (Table 3). The majority 73% (n = 539) reported that they had knowledge about the reports of side effects for HPV vaccine, and over half of the school nurses (56%, n = 409) agreed to the statement that they needed more education (training) about HPV.

**Table 3:** School nurses’ perceived knowledge about HPV vaccination.

	Strongly agree n (%)		Partially agree n (%)		Partially disagree n (%)		Strongly disagree n (%)		P value†
	2016	2013	2016	2013	2016	2013	2016	2013	
I have good knowledge about HPV to inform girls	454 (61.7)	529 (62.2)	264 (35.9)	226 (26.6)	7 (1.0)	96 (11.3)	0 (0)	0 (0)	0.154
I have good knowledge about HPV to inform parents	398 (54.1)	481 (56.5)	315 (42.8)	296 (34.8)	10 (1.4)	61 (7.2)	0 (0)	13 (1.5)	0.629
I have good knowledge to answer parents’ questions about HPV vaccine	292 (39.7)	351 (41.2)	415 (56.4)	433 (50.9)	20 (2.7)	24 (2.8)	1 (0.1)	2 (0.2)	0.275
I have good knowledge about the safety profile of HPV vaccine*	281 (38.2)	-	419 (56.9)	-	27 (3.7)	-	1 (0.5)	-	-

^†^Mann-Whitney *U* test

*This question was added to the 2016 survey

### Information about HPV vaccine to parents and pupils

There was significant difference regarding the information provided to the girls before vaccination (Table 4). More nurses (59% in 2016 vs 52% in 2013) informed girls only separately, while there were no differences in information given to boys and girls together or information provided only to boys. In addition, there were no differences in information provided to parents. For more information, see Table 4.

**Table 4:** Information given by school nurses’ about HPV vaccine to parents and pupils.

	Yes, n (%)		No, n (%)		P-value†
	2016	2013	2016	2013	
Written information to parents	717 (97.4)	828 (97.3)	10 (1.4)	23 (2.7)	0.066
Information at parental meetings	125 (17.0)	175 (20.6)	602 (81.8)	676 (79.4)	0.089
Information by email or telephone on parents’ initiative	233 (31.7)	290 (34.1)	494 (67.1)	561 (65.9)	0.394
Oral information about HPV vaccine to boys and girls	250 (34.0)	286 (33.6)	478 (64.9)	565 (66.4)	0.009
Oral information about HPV vaccine to girls	433 (58.8)	442 (51.9)	295 (40.1)	409 (48.1)	<.001
No oral information about HPV vaccine to boys or girls	91 (12.4)	123 (14.5)	637 (86.5)	728 (85.5)	0.005

^†^Pearson Chi-Square

#### Questions and concerns from parents about HPV vaccine

We found that more school nurses had been contacted by parents who had questions about the vaccine, 76% in 2013 compared to almost 90% in 2016. In total, 465 (63%) agreed strongly and 194 (26%) agreed partially to this statement. In the open-ended questions, 83% (n = 610) reported that they had received (specific) questions from parents. Most questions were related to vaccine safety, and the most frequently asked questions from parents (81%, n = 493) concerned side effects.

‘Are you absolutely sure that the vaccine has no side effects?’

‘Dare I give my daughter the vaccine? What are the risks?’

Other commonly asked questions were regarding the daughters’ age (13%, n = 66) and if it was possible to postpone the vaccination and vaccinate the daughter later.

‘Why it is given to such young girls who often have several years left before sexual debut?’

‘Can we wait a year? She is too young.’

Some nurses reported a diverse area of frequently asked questions among parents such as do you need a booster dose (n = 7), why should you choose to vaccinate (n = 2), why are boys not included and why are only two doses distributed (n = 2). Moreover, some school nurses (n = 20) reported that the most frequently asked question was *what is your opinion as a school nurse*, *should we vaccinate our daughter or not*? ‘What would you [as the school nurse] do? ‘

### Financial support by the government

Financial support by the government has been used to cover the extra expenses incurred by HPV vaccinations to a significantly (p <.001) higher degree compared with in 2013. Nearly four out of ten school nurses (38.5%, n = 283) reported that the financial support had been used for an additional nurse, 44 (6.0%) for educational training (no one reported this in 2013), and nine nurses (1.2%) for extra work hours. However, 200 nurses (27.2%) reported that they did not know how or if the financial support had been used for HPV vaccinations. One of five nurses (20.2%, n = 153) reported that as far as they knew, financial support had not been used to cover the extra expenses incurred as a result of HPV vaccinations.

## Discussion

As hypothesised, school nurses had more favourable attitudes towards the vaccination programme and perceived less barriers compared with in 2013. However, most nurses were contacted by parents who had questions and concerns about the vaccine. Contrary to our hypotheses, there were no differences in perceived knowledge about HPV vaccine compared with in 2013. In addition, the majority agreed that they needed more education about HPV.

It is encouraging that the nurses have a more favourable attitude towards the national HPV vaccination programme. The main reason to the more favourable attitude is probably because of the reduced workload. Since 2015, two doses are distributed with a time interval of six months instead of three doses given within six months. School nurses’ positive attitudes can also be explained by their longer experience of administrating the vaccine and therefore they are more comfortable to offer a safe vaccine [22]. In addition, the governmental financial support is used to cover the expenses for HPV vaccinations to higher degree compared to at the start of the vaccination programme.

The majority have confidence in decisions made by the authorities about HPV vaccination. Trust in governmental recommendation and trust in recommendations from healthcare providers are important factors for acceptance of HPV vaccinations [7, 23–25]. More nurses agreed that boys also should be included in the school based vaccination programme, which is in line with the findings in our recent qualitative study about parents’ views to also include boys in the national vaccination programme [26]. This is encouraging since there is currently a discussion about including boys too in the national school based HPV vaccination programme.

The finding that more nurses agreed that those who declined vaccination should be offered the vaccination later on is an important finding. School nurses were recently assigned to offer HPV vaccination to those who had missed the opportunity or to those who previously have declined the vaccine. HPV vaccination was initially “a onetime offer” in Sweden, and this second chance is an open window that needs to be endorsed. This is beneficial for the individual as well as for the public health in order to decrease the burden of HPV. School nurses’ favourable attitudes towards including boys and to offer the vaccine up to age18 can reflect the role of school nurses as advocates for children’s health; their assignment is to promote equal health and decrease health disparities [16, 27]. However, even though these results are encouraging, it should be emphasised that school nurses need time and adequate resources since the vaccinations are time-consuming [10, 13, 18]. This is crucial since Sweden has a large amount of newly arrived immigrants and refugees, and this vulnerable group need special support.

Although the Swedish national HPV vaccination programme in many ways is well functioning, there are some challenges. Almost all nurses had been contacted by parents who had questions and concerns about the vaccine, especially regarding vaccine safety. Fear of side effects was reported as being the most common reason for parents to decline the vaccine. This is not surprising since there has been massive media debate about the safety aspects of HPV vaccine [22]. Rumours and misconceptions as well as reports of side effects, such as Postural orthostatic tachycardia syndrome (POTS) and Postural orthostatic tachycardia syndrome (CRPS), can affect national vaccination programmes as reported in Denmark and Japan, where there has been a sharp decline in the vaccine uptake, from over 70% to about 27% and 1%, respectively [22, 28]. Misconceptions can easily spread on the Internet and social media and contribute to parents’ concerns over the vaccine [29, 30]. However, the European Medical Agency confirms that the evidence does not support a causal link between the vaccines (Cervarix, Gardasil/Silgard and Gardasil 9) and development of CRPS or POTS. Therefore, there is no reason to change the recommendations to vaccinate against HPV [22].

More than half of the nurses (56%) agreed that they needed more education (training) about HPV. Moreover, not even four out of ten believed that they had good knowledge about the safety profile of HPV vaccine. In order to meet the need for adequate knowledge about HPV vaccine, the Public health agency in Sweden has recently updated the educational material dispersed to school nurses [31]. It is essential that the providers, i.e. the nurses, receive adequate education and training in order to feel confident to address parents’ frequently asked questions and concerns. McRee et al. [32] emphasise the importance of strengthening healthcare providers’ self-efficacy in order to address parents’ vaccine hesitancy. One way to strengthen the nurses’ self-efficacy is by providing continuous education and training about HPV and HPV vaccine. We have previously found that school nurses who perceive higher level of knowledge about HPV vaccine have more favourable attitudes towards the vaccination programme compared to school nurses with lower perceived knowledge [18]. This is in line with Rosen et al. [10, 11] and Zimet et al. [33], emphasising school nurses and healthcare providers’ need for adequate knowledge about HPV. Consequently, knowledge is an essential factor and most likely, school nurses who perceive sufficient knowledge about HPV feel more at ease in addressing parents’ questions and concerns.

Besides knowledge of HPV, studies also highlight the importance of healthcare professionals’ communication skills [6, 14, 33, 34]. In communications with parents, school nurses should emphasise that HPV vaccine is safe and highly efficient in protecting against HPV-related diseases, especially when distributed to children before exposure to HPV. Addressing parents’ questions and concerns might put at ease parental vaccine hesitancy, which might lead to increased vaccine coverage.

### Strengths and limitations

A large number of school nurses from all counties and regions participated, representing a diverse population working in different socioeconomic areas, and in both public and private schools. Consequently, the results are likely to be representative of the Swedish school nurses vaccinating against HPV. However, one limitation is that at the time of the study, we could not get reliable data of the exact number of school nurses vaccinating against HPV in Sweden. The number is most likely comparable with the number in 2013, approximately 1,050 nurses. Thus, slightly fewer school nurses (n = 736) completed the questionnaire than previously (n = 851) [18]. One reason for this might be that at the time of the present study, school nurses had a very high workload due to extraordinary conditions. In 2015–2016, Sweden accepted more refugees per capita than any other European country. Many of these refugees were unaccompanied minors, requiring extra healthcare such as additional vaccinations provided by the school nurse. Another possible limitation is the lack of information about non-respondents. However, we believe that the participating nurses are representative of the Swedish school nurses, and that the results can be generalised.

## Conclusions and clinical implications

Although the Swedish vaccination programme against HPV in many ways is well functioning, there is room for improvement. School nurses have a more favourable attitude towards the vaccination programme against HPV compared to at the start of the national vaccination programme in 2012. A high proportion of nurses had been contacted by parents with diverse questions and concerns. The nurses believed that they needed more education about HPV. Therefore, it is essential to provide ongoing education and training for school nurses who are key healthcare professionals for providing information about HPV to parents and to pupils.

To overcome some of the remaining barriers, school nurses need adequate resources and training for managing the complex task of vaccinating against HPV. Parents have questions and concerns about vaccine safety. Thus, there is an urgent need for interventions with the aim to increase parents’ knowledge about HPV and HPV vaccine in order to increase vaccine uptake, especially in areas with low coverage. The school nurse is a key person for information sharing about HPV; thus, educational interventions might be delivered by the school nurse at parental meetings when time comes for parents’ decision about HPV vaccination for their child.

## Supporting information

S1 File STROBE checklist. (DOCX)

S2 File Data set 2016. (XLS)

S3 File Data set 2013. (XLS)
