# Supplementary material for: School nurses’ attitudes towards and experiences of the Swedish school-based HPV vaccination programme – A repeated cross sectional study
Source: PLoS One. 2017 Apr 18;12(4):e0175883. doi: 10.1371/journal.pone.0175883 (PMC5395208; doi:10.1371/journal.pone.0175883)
Supplement: S1 File — (DOCX) [file pone.0175883.s001.docx]

**STROBE 2007 (v4) Statement—Checklist of items that should be included in reports of *cross-sectional studies***

| **Section/Topic** | Item # | Recommendation | Reported on page # |
| --- | --- | --- | --- |
| **Title and abstract** | 1 | (*a*) Indicate the study’s design with a commonly used term in the title or the abstract | Title page, page 1 |
|  |  | (*b*) Provide in the abstract an informative and balanced summary of what was done and what was found | Abstract, page 2 |
| Introduction | | |  |
| Background/rationale | 2 | Explain the scientific background and rationale for the investigation being reported | Pages 3-4 |
| Objectives | 3 | State specific objectives, including any prespecified hypotheses | Page 4 |
| Methods | | |  |
| Study design | 4 | Present key elements of study design early in the paper | Page 5 |
| Setting | 5 | Describe the setting, locations, and relevant dates, including periods of recruitment, exposure, follow-up, and data collection | Pages 5-6 |
| Participants | 6 | (*a*) Give the eligibility criteria, and the sources and methods of selection of participants | Page 5 |
| Variables | 7 | Clearly define all outcomes, exposures, predictors, potential confounders, and effect modifiers. Give diagnostic criteria, if applicable | Page 6 |
| Data sources/ measurement | 8* | For each variable of interest, give sources of data and details of methods of assessment (measurement). Describe comparability of assessment methods if there is more than one group | Page 6 |
| Bias | 9 | Describe any efforts to address potential sources of bias | Pages 5-6 |
| Study size | 10 | Explain how the study size was arrived at | Pages 5-6, 18-19 |
| Quantitative variables | 11 | Explain how quantitative variables were handled in the analyses. If applicable, describe which groupings were chosen and why | Pages 6-7 |
| Statistical methods | 12 | (*a*) Describe all statistical methods, including those used to control for confounding | Pages 6-7 |
|  |  | (*b*) Describe any methods used to examine subgroups and interactions | N/A |
|  |  | (*c*) Explain how missing data were addressed | All items had response rates of ≥98%. The internal missing data were less than <2%, thus we did not address this in the analysis.  The external validity is discussed in Strengths and Limitations on pages 18-19. We could not get reliable data on the number of school nurses vaccinating against HPV in the school year 2015/2016. |
|  |  | (*d*) If applicable, describe analytical methods taking account of sampling strategy | N/A |
|  |  | (*e*) Describe any sensitivity analyses | N/A |
| **Results** |  |  |  |
| Participants | 13* | (a) Report numbers of individuals at each stage of study—eg numbers potentially eligible, examined for eligibility, confirmed eligible, included in the study, completing follow-up, and analysed | As mentioned earlier, this is discussed in Strengths and Limitations on pages 18-19. |
|  |  | (b) Give reasons for non-participation at each stage | N/A |
|  |  | (c) Consider use of a flow diagram | N/A |
| Descriptive data | 14* | (a) Give characteristics of study participants (eg demographic, clinical, social) and information on exposures and potential confounders | Table 1, page 8 |
|  |  | (b) Indicate number of participants with missing data for each variable of interest | See tables 2-4, all items have response rates above 98%. |
| Outcome data | 15* | Report numbers of outcome events or summary measures | See tables 2-4 and Result pages 7-15. |
| Main results | 16 | (*a*) Give unadjusted estimates and, if applicable, confounder-adjusted estimates and their precision (eg, 95% confidence interval). Make clear which confounders were adjusted for and why they were included | Results pages 7-15 |
|  |  | (*b*) Report category boundaries when continuous variables were categorized | Results pages 7-15 |
|  |  | (*c*) If relevant, consider translating estimates of relative risk into absolute risk for a meaningful time period | N/A |
| Other analyses | 17 | Report other analyses done—eg analyses of subgroups and interactions, and sensitivity analyses | Results pages 7-15 |
| Discussion |  |  |  |
| Key results | 18 | Summarise key results with reference to study objectives | Pages 15-16 |
| Limitations | 19 | Discuss limitations of the study, taking into account sources of potential bias or imprecision. Discuss both direction and magnitude of any potential bias | Pages 18-19 |
| Interpretation | 20 | Give a cautious overall interpretation of results considering objectives, limitations, multiplicity of analyses, results from similar studies, and other relevant evidence | Pages 15-18 |
| Generalisability | 21 | Discuss the generalisability (external validity) of the study results | Pages 18-19 |
| Other information |  |  |  |
| Funding | 22 | Give the source of funding and the role of the funders for the present study and, if applicable, for the original study on which the present article is based | This information is provided via Plos One submission system. |

*Give information separately for cases and controls in case-control studies and, if applicable, for exposed and unexposed groups in cohort and cross-sectional studies.

**Note:** An Explanation and Elaboration article discusses each checklist item and gives methodological background and published examples of transparent reporting. The STROBE checklist is best used in conjunction with this article (freely available on the Web sites of PLoS Medicine at http://www.plosmedicine.org/, Annals of Internal Medicine at http://www.annals.org/, and Epidemiology at http://www.epidem.com/). Information on the STROBE Initiative is available at www.strobe-statement.org.
